# Green synthesis of polyherbal silver nanoparticles from *Cassia fistula* and *Manilkara zapota*: characterisation and evaluation of wound-healing potential

**DOI:** 10.1016/j.jgeb.2026.100826

**Published:** 2026-09-28

**Authors:** Archana Hari, Shobana Sampath, B. Vijayageetha, Douglas Law, Gholamreza Abdi

**Affiliations:** a Department of Biotechnology, SRM Institute of Science and Technology (E&T), Ramapuram Campus, Chennai, Tamilnadu, India; b Department of Biotechnology, Vel Tech Rangarajan Dr Sagunthala R&D Institute of Science and Technology, Chennai 600062, Tamil Nadu, India; c Department of Biotechnology, Rajalakshmi Engineering College (Autonomous), Affiliated to Anna University, Thandalam, Chennai, Tamil Nadu 602 105, India; d Faculty of Health and Life Sciences, INTI International University, Nilai, 78000, Negeri Sembilan, Malaysia; e Faculty of Health Sciences, Shinawatra University, Pathum Thani 12160, Thailand; f Department of Biotechnology, Persian Gulf Research Institute, Persian Gulf University, Bushehr,75169, Iran

**Keywords:** Wound healing, *Cassia fistula*, *Manilkara zapota*, Polyherbal, Silver nanoparticle, Human health

## Abstract

In this study, methanol concentrate of flowers and leaves of *Cassia fistula* and leaves of *Manilkara zapota* was used to obtain silver nanoparticles (AgNPs), followed by evaluation of wound healing potential in an animal model. The objective was to assess the overall wound-healing potential of the plant-mediated AgNP formulation rather than to compare different synthesis routes. In SEM analysis, particle dimensions were irregular, ranging from 20 nm to 50 nm. In FTIR, distinct bond stretching was observed at 3332.16, 2114.20, 1635.70, and 637.27. The antibacterial assay of plant Ag NPS in the case of *Staphylococcus aureus* MTCC 96 showed its maximum zone of inhibition at 17 mm. Accordingly, the antibacterial activity reported in the present study is restricted to *S. aureus* MTCC 96, the bacterial strain experimentally evaluated and no other pathogenic bacteria have been experimentally. In the excision wound model, PSN (polyherbal silver nanoparticle) and PCE (polyherbal crude extract) at a concentration of 5% *w*/w showed wound contraction of 75.58% in group IV rats and 70.38% in group III rats. The results were higher when compared with group II. Cytokine TNF-α in blood serum was low in group IV in comparison to group II at the post-wound treatment. It was reported 6.49 pg/mg concentration of TNF-α for group IV and 12.69 pg/mg concentration of TNF-α for group II at the 14th day, indicating that TNF-α is an inflammatory cytokine that is found to be released at the early stage of the wound healing. Histopathology of the group III and IV-treated tissue showed better epithelization than group II rats. Thus, PSN confirmed its significant levels in wound healing studies, which will show its potential in skin care and treating skin-related issues.

## Graphical abstract

**Figure:**
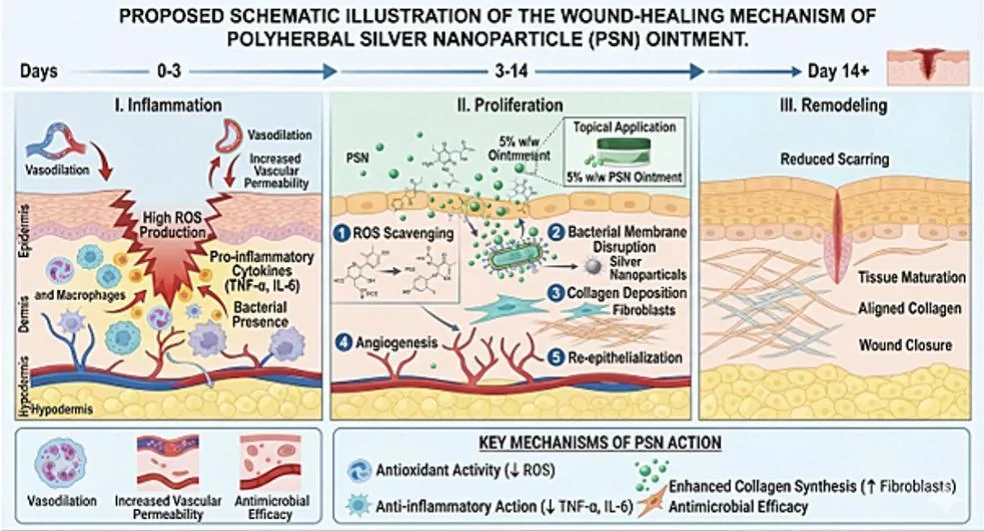

## 1 Introduction

Wound healing is a complex, finely coordinated biological cascade divided into four overlapping phases: hemostasis, inflammation, proliferation, and tissue remodeling. Disruptions at any stage, often exacerbated by persistent microbial infection or excessive local oxidative stress, can stall tissue repair and lead to chronic, non-healing wounds. Recent work has emphasized the pivotal role of cellular and molecular frameworks, noting that uncontrolled reactive oxygen species (ROS) destroy extracellular matrices and cause severe cellular senescence at the injury site, as outlined in a comprehensive review in Communications Biology.^1^ Consequently, modern therapeutic interventions increasingly focus on multi-target systems that simultaneously eliminate bacterial bio-burden and clear excess localized free radicals.

To address these dual challenges, biogenic nanotechnology has emerged as a frontline strategy. Green-synthesized silver nanoparticles (AgNPs) utilize plant-derived secondary metabolites as natural reducing and capping agents, offering a sustainable alternative to hazardous chemical synthesis. According to recent breakthroughs published in *Nano Biomedicine and Engineering,*^2^ the phenolic, alkaloid, and carbonyl groups present in botanical extracts not only drive the reduction of silver ions but also embed secondary therapeutic traits like inherent antioxidant and anti-inflammatory properties into the resulting nanostructures. This multi-functional synergy is supported by recent findings in *Discover Nano,*^3^ which confirmed that plant-mediated nanoparticle systems display exceptional biocompatibility and significantly lowered dermal cytotoxicity profiles compared to traditional chemical alternatives.

Integrating these green-synthesized nanoparticles into topical matrices allows for controlled, localized drug delivery directly within the compromised skin layers. During the inflammatory-to-proliferative transition, modulating localized signaling biomolecules is essential to prevent chronic wound extension. As reported in *Emergent Materials,*^4^ topical biomaterial platforms mitigate prolonged inflammation by rapidly down-regulating pro-inflammatory markers (such as tumour necrosis factor-alpha TNF-alpha and interleukin-1 beta IL-1), while concurrently up-regulating angiogenic markers like CD31 to foster rapid neovascularization. Furthermore, research in the *European Polymer Journal*^5^ demonstrates that establishing an optimal microenvironment accelerates keratinocyte migration and dermal fibroblast proliferation, leading to complete structural closure and uniform re-epithelialization across boundary gaps.

Further the silver nanoparticles underwent an antibacterial study against the desired organism. This nanoparticles with the effectiveness of the synergistic extract are combined with the silver nanoparticles for the future wound dressing.^6^ Over the world, several herbs that were discovered to have therapeutic properties are still employed in traditional medicine to treat illnesses or syndromes. It was found that traditional medicine practitioners served as the primary healthcare providers in rural communities.

The phytochemicals present in plants make them unique for their unique medicinal properties, they include alkaloids, tannins, flavonoid, terpenoids, phenolic compounds steroids, resins, and other secondary metabolites.^7^ These substances were biologically reactive and had a big impact on infections and parasites. Many bioactive phytochemicals in plants admired for the ability to fight against the microbes and boosting the immune system of the body.^8^ Restoring the skin's characteristics and disrupted anatomical continuity required the healing of wounds. There are four phases to the intricate and complex process of wound healing: hemostasis, inflammation, proliferation, and remodeling.^9^ Chronic wounds were often referred to as ‘difficult to heal’ as they prolong in duration taken (4 to 12 weeks) for recovery. Roughly 1% of people on the planet are thought to have wounds that are difficult to heal and are linked to a lower quality of life.^10^

Historically, a variety of formulations for the treatment of burns, wounds, cuts, and skin disorders have been made using plants.^11^ Through a variety of methods, the isolates derived from plants promote tissue regeneration and repair; these effects frequently work in concert to promote total healing. Numerous plants have been employed in conventional medicine as anti-inflammatory, wound-healing, and anti-allergic medicines because they contain therapeutic amounts of flavonoids.^12^

In medicine, nanotechnology has been used to develop therapeutic medication delivery systems and cures for a wide range of illnesses and conditions.^13^ So, there were various types of nanocarriers which were under clinical trials and some of them are used as nanomedicines for targeted drug delivery such as nanoparticles, nano spindles, nanofibers, nanotubes, nanosphere, nanorods, nano dendrimers, nanocrystals etc.,.^14^ In particular, green-synthesized silver-based nanoparticles have attracted attention because of their reported antimicrobial, antioxidant and anti-inflammatory activities and their potential applications in wound care and other biomedical fields.^5^, ^15^, ^16^, ^17^

While individual extracts of *Cassia fistula* and *Manilkara zapota* possess documented therapeutic properties, their consolidated evaluation in a polyherbal silver nanoparticle (PSN) ointment matrix remains largely unexplored. This study investigates the dual-action synergy of a 5% (*w*/w) biogenic PSN formulation. By tracking its capacity to regulate local antioxidant enzyme profiles (SOD, CAT, and LPO), suppress serum TNF-alpha concentrations, and accelerate cellular gap closure via in vitro scratch assays, this work provides a robust framework for scalable, plant-mediated nanomedicine in advanced wound care management.

## 2 Materials and methods

### 2.1 Plant material

Fresh leaves of *Manilkara zapota* along with leaves and flowers of *Cassia fistula* were collected from Aranvayulkuppam, Thiruvallur, Tamil Nadu. After being separated, the leaves were properly cleaned with tap water, allowed to dry in the shade, ground into a fine powder, and then sealed in airtight bottles. With solvents based on their polarity, such as methanol and aqueous ethanol (1:1), the powdered form of the leaves was successively extracted using the percolation method. Using a rotary evaporator, the extracted substances were concentrated and solvent-freed at low pressure. Prior to being employed for analysis, the dried-out crude extracted substances were sealed in airtight bottles and refrigerated after being weighed to determine the extractive yield.

### 2.2 Extraction method

Using methanol as a solvent, the powdery residue of the plant's leaves and flowers was extracted one after the other by the percolation process.^18^ Using a rotary evaporator, the extracts have been concentrated and solvent-freed at low pressure. Airtight bottles containing the calibrated crude extract were kept in the refrigerator.^19^

### 2.3 Synergism of plant extract and synthesis of silver nanoparticle

Leaves from *Manilkara zapota*, along with flowers from *Cassia fistula*, were collected in equal proportions (i.e., 1:1:1).^20^ The plant silver nanoparticle was prepared by mixing aqueous solution of silver nitrate (1 mM) and fresh methanol extract of the plants. The present study used a fixed experimental condition consisting of 1 mM AgNO₃, a 1:1:1 plant extract ratio, a 1:9 AgNO₃-to-extract volume ratio, and 24 h incubation at room temperature in the dark. The solution was incubated overnight in the dark at room temperature for 24 h.^21^ After 24 h, the colourless silver nitrate solution turned pale yellow to reddish brown. The synergistic plant-mediated AgNPs were centrifuged for 20 mins at 5000 rpm. The pellets were allowed to air-dry while the supernatant was disposed of.^22^

### 2.4 Characterisation of nanoparticles

The depletion of silver metal ions to silver nanoparticle was analysed by using UV- visible Spectrophotometer. Using a Perkin Elmer Lambda 25 spectrometer, the absorption maxima were scanned over the 300–800 nm wavelength range to conduct the UV–vis analysis. The aqueous component was sampled at various intervals.^23^ FESEM was used to characterise the nanoparticles' size and shape using Carl Zeiss FESEM Sigma360 (Schottky FEG) with an Oxford EDS detector. FTIR measurements were performed to identify functional groups and putative biomolecules associated with the reduction and surface stabilization of the synthesized silver nanoparticles.^24^, ^25^

### 2.5 Screening of antibacterial activity

After preparing the nutrient agar, it was added to the sterile petri plates and let to solidify. On it, a 24-h *Staphylococcus aureus (MTCC 96)* bacterial culture was swabbed.^26^ Using sterile forceps, the test sample at various concentrations was put over the agar plate. The control was 25 μL of tetracycline. After that, the plates were incubated for 24 h at 37 °C. The inhibitor's diameter was measured after incubation, and the units are given in mm.^27^

### 2.6 Anti-oxidant assay

On day 14, under anaesthesia, tissues from the wounds were collected.

#### 2.6.1 Assay of lipid peroxidation (LPO)

Thiobarbituric acid (TBA) was used to assess malondialdehyde (MDA), a byproduct product of lipid peroxidation, by producing a coloured material in the reaction. The coloured line was read at 532 nm.^28^

#### 2.6.2 Assay of superoxide dismutase

By tracking the oxidation of adrenaline, the activity of SOD was determined. The tubes were filled with 0.5 mL of EDTA (0.6 mm) solution and 2.5 mL of carbonate-bicarbonate buffer (0.3 m, pH 10.2). A UV spectrophotometer was used to measure the change in absorbance after injecting 0.5 mL of epinephrine (1.8 mm) at 420 mm for two minutes at intervals of 15 s. The samples from groups I, II, and IV were suitably diluted. Protein units, minutes, and grammes were used to express the activity.^29^

#### 2.6.3 Assay of catalase

(nmoles of hydrogen peroxidase consumed /min/mg of protein) Using spectrophotometry, the rate of hydrogen peroxidase breakdown was determined at 240 nm.^30^

### 2.7 Invitro scratch assay

Based on scratch wound healing assay, the procedure was followed and the results were obtained for every 24 h.^31^

#### 2.7.1 Excision wound model

To oversee and regulate animal research and secure clearance from the Institutional Animal Ethics Committee, this study was carried out in accordance with the board's animal ethics rules (IAEC) of C.L. Baid Metha College of Pharmacy, Chennai, Tamil Nadu with approval No: 06/321/PO/Re/S/01/CPCSEA/dated 31/03/19.

For the surgical excision wound model, four groups of six rats each were employed. The animals in Group I received no therapy; they were used as an untreated negative control. Group II animals were treated with standard drug ointment povidone iodine. Group III were treated with crude polyherbal formulation and group IV were treated with polyherbal silver nanoparticle.

All animal protocol were performed and approved by the ethical committee. All rats were treated humanely throughout the experiment. On day 0, the rat's dorsum was shaved and cleaned by the saline soaked gauze and 70% ethanol solution. A permanent marker was used to create a 314 mm^2^ circular mark.^32^ Prior to wound creation and tissue collection, all animals were anaesthetized using ketamine (80 mg/kg, i.p.) and xylazine (10 mg/kg, i.p.) to ensure deep anaesthesia and minimize distress.

Standard ointment povidone iodine 5% *w*/w and 5% *w*/w of polyherbal solution (ointment base) and polyherbal silver nanoparticle (ointment base) was applied to the respective group of rats. This was considered as day 0. On experimental observation, all animals were housed in their cages individually and ambient temperature was maintained with proper food and water. The time it took for epithelialisation to occur and the percentage of wound contraction were used to measure the wound-healing properties of crude extract (PCE) and polyherbal silver nanoparticle formulation (PSN).^33^

Percentage of wound contraction calculated for PCE and PSN formulation is as follows:

$$\%\text{Wound contraction}=\frac{\left(\text{wound area}\;\mathrm{on}\;0\mathrm{th}\;\mathrm{day}-\text{wound area}\;\mathrm{on}\;\mathrm{nth}\;\mathrm{day}\right)\;}{\text{wound area}\;\mathrm{on}\;0\mathrm{th}\;\mathrm{day}}*100$$

where n = the days when measurement was taken.^34^

### 2.8 Measurement of TNF-α concentration

The mouse TNF-α in blood serum was quantified in vitro using the RayBio Mouse TNF-α ELISA (Enzyme-Linked Immunosorbent Assay) kit. Mouse TNF-α coated in an antibody specific manner on a 96-well plate is used in this test. The wells were filled with standards and blood serum, and the immobilised antibody bound the TNF-α in the serum to the wells.^35^ Following a washing, biotinylated anti-Mouse TNF-α antibody was applied to the wells. The wells were filled with HRP-conjugated streptavidin after the unbound biotinylated antibody had been rinsed. Following another washing, the wells were filled with a TMB substrate solution, which produced a hue corresponding to the amount of TNF-α bound. The stop solution caused the colour to change from blue to yellow, and the intensity was recorded at 450 nm.^36^

### 2.9 Histological analysis

The recovered wound tissue underwent thorough histological processing. The tissue was preserved in formalin, cut, and processed—that is, it was cleaned in xylene, dehydrated in graded alcohol, and embedded in paraffin at 58 °C to 60 °C.^37^ These tissue blocks were divided into 4–5 μ thick sections, stained with eosin and haematoxylin, and then digitally photocopied and mounted. After that, slides were examined under a light microscope, and pictures were taken.^38^

### 2.10 Statistical analysis

A statistical software program that is commercially accessible was used to analyse the data (mathcracker/one-way-anova software). To determine the significance of differences between the control and PCE and PSN-treated rats, computations were done. Results were presented as mean ± SD and the *p* value less than 0.001, 0.01 and 0.05 were considered to be statistically significant.^39^

## 3 Results

Plant extracts contain polysaccharides and phytocomponents. The speed of the reaction reducing Ag^+^ ions in the plant extract and the change in colour intensity are the main objective for the synthesis of silver nanoparticles. In Fig. 1, distinct change in the colour of the sample with standard concentration of AgNO_3_ was observed, but the colour of the control (crude plant extract) remained un-changed. The colour of the sample turned light brown, which became deeper after 24 h. Prior work of other authors has also reported similar colour change due to silver nanoparticle formation. The result of the synthesis of silver nanoparticles with *Manilkara zapota* seed extract, clearly specify the development of the silver nanoparticles. The colour of sample turned light brown, which became deeper after 24 h. After characterisation studies, the formulation was then used to treat the wound. Prior work of other authors has also reported similar colour change due to silver nanoparticle formation.

**Fig. 1:**
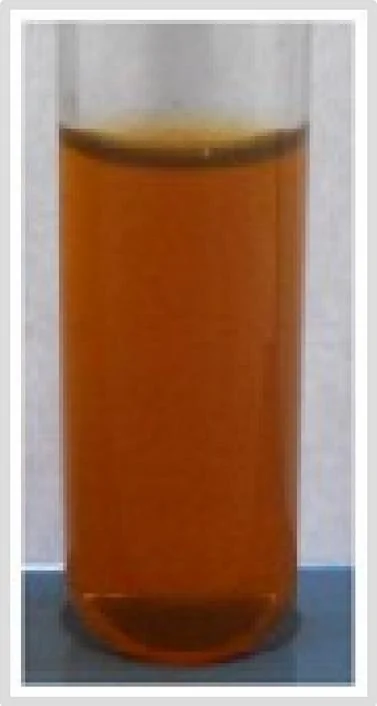
Synthesis of plant silver nanoparticles. Colour change in the tube after 24 h of the reaction time.

With the help of UV–Visible spectroscopy, we examined the absorption peak of the nanoparticles. The maximum absorption peak was noted at 425 nm (Fig. 2), and the synthesized silver nanoparticles showed a surface plasmon resonance (SPR) band within the 423–438 nm range. This observation is consistent with the optical behaviour reported for biosynthesized silver nanoparticles, for which UV–visible spectroscopy has been used to confirm nanoparticle formation.^40^ The spectra of silver nanoparticles synthesized using leaf and callus-based extracts have also been reported in the 440–445 nm range.^41^

**Fig. 2:**
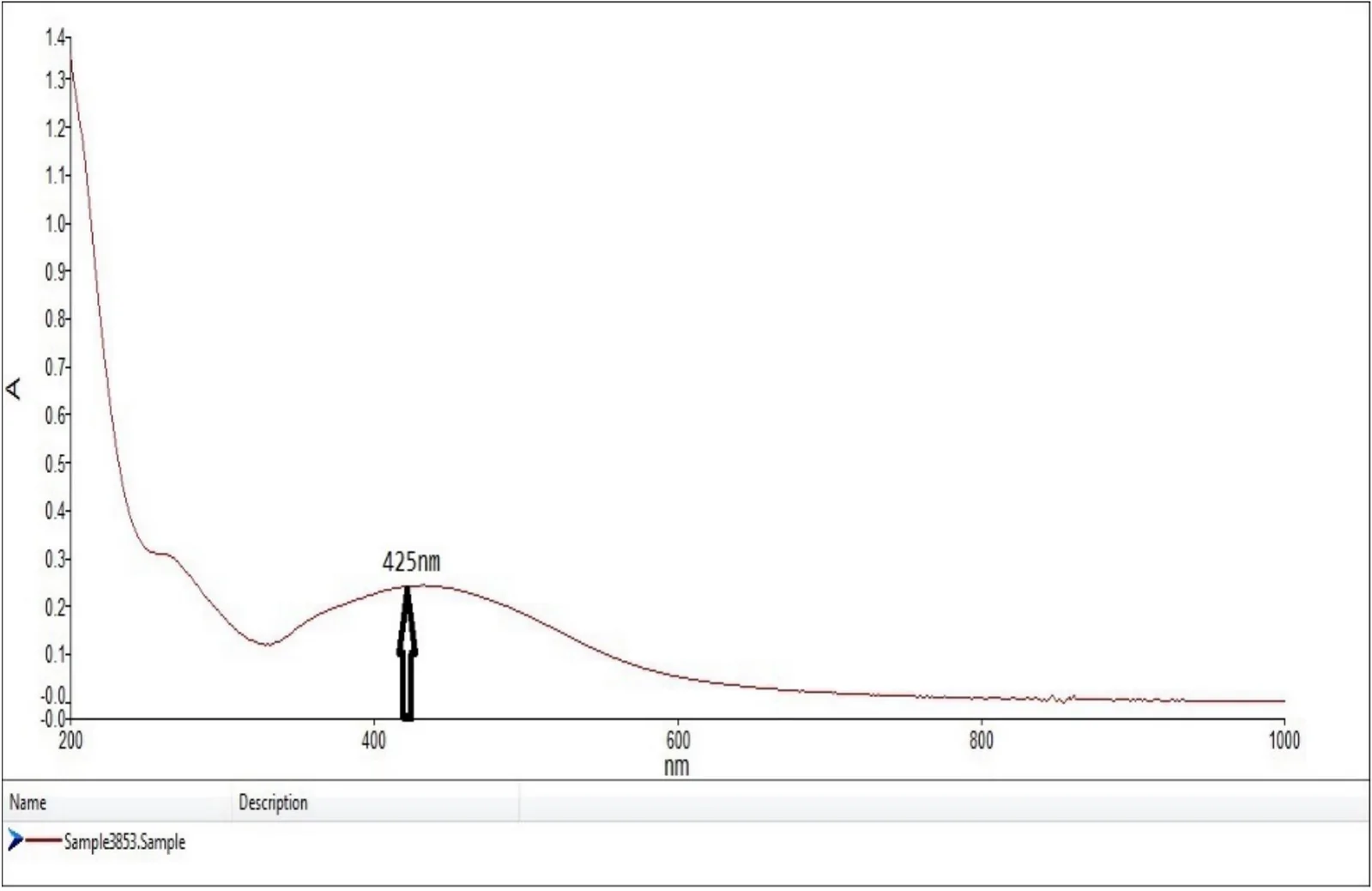
UV–visible spectroscopy of silver nanoparticles by methanol extract of *Cassia fistula* and *Manilkara zapota.* Absorption peak was noted to be at 425 nm.

FESEM is a quantitative analysis where the size of the particles can be conducted. It provides images of nanoparticles and their agglomerates in sufficient solution. SEM analysis shows high-density AgNPs synthesized by the synergistic herbal formulation. The figure depicts the SEM characterisation of synergistic plant nanoparticles (*Cassia fistula* flowers and leaves of *Manilkara zapota* leaves in the ratio 1:1:1) in the magnification range of 30,000×. Particles morphological structure, where the SNP are of irregular in size (Fig. 3). They were equally dispersed in and around. The observed size of the nanoparticles ranged from 20 nm - 50 nm. It was shown that relatively irregular spherical. Synthesis parameters such as pH and temperature can significantly influence the nucleation, growth, and aggregation of biologically synthesized nanoparticles; therefore, optimization of these parameters is necessary to determine whether a more uniform and homogeneous nanoparticle morphology can be achieved. AgNPs were formed and the characterisation appears to have more pores. SEM image of silver nanoparticles described the surface topography and the composition of the sample. The biomolecules present in the nanoparticles confirm the presence of the plant's metabolites in the extract which is responsible for stabilizing the silver nanoparticle. In an inquiry on cytotoxic effect of bioactive AgNPs synthesized using *Cassia fistula* flower extract on breast cancer cell MCF-7, the FE-SEM outcomes were 50 nm and the shape of the silver nanoparticles were spherical. Our synergistic plant silver nanoparticles do fall in around this range which clearly states that *Cassia fistula* with the other plants can improve its efficiency in any translational medicines.^42^ EDAX Spectrum confirmed the presence of silver peaks.

**Fig. 3:**
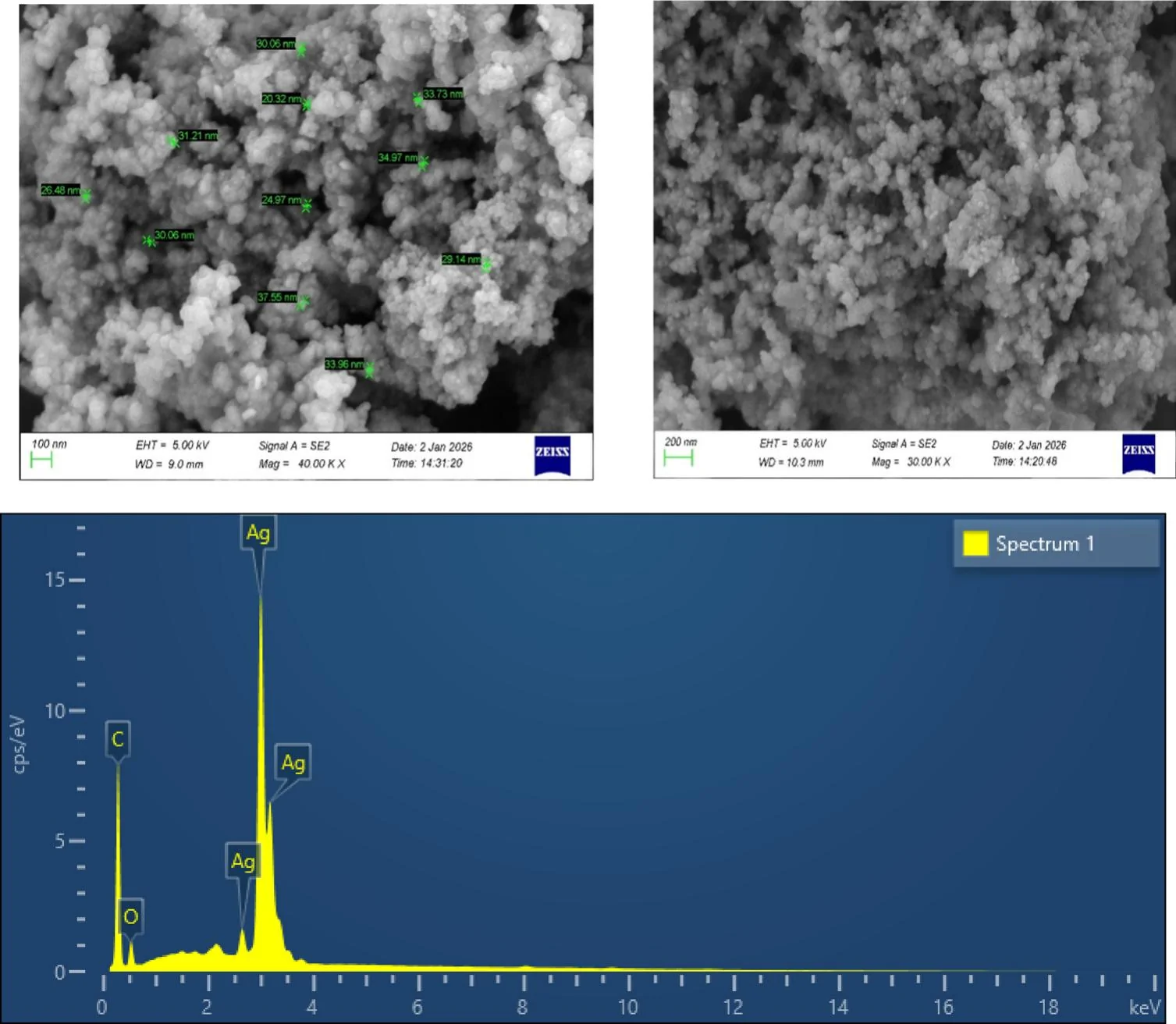
Scanning electron micrograph of silver nanoparticles by methanol extract of *Cassia fistula* and *Manilkara zapota.* Good dispersion of the nanoparticle in the solution and size ranges from 20 nm - 50 nm.

The local molecular environment of the capping agents on the silver nanoparticles and the chemical makeup of their surface could both be investigated using FTIR spectroscopy. Results of FTIR analysis of this study showed different stretch of bonds shown at different peaks; 3332.16 which showed the alcohol, aliphatic primary amine, and secondary amine compound group frequencies of. “polymeric” O—H stretch demonstrating the reducing agent's hydroxyl group presence. 2114.20 showed frequencies of C triple bond C indicating the presence of alkyne group. 1635.70 alkene group frequencies of alkyl C=C stretch. 637.27 halo compound frequencies of C—Br stretch and C—Cl stretch. Thus FTIR spectrum obtained in the present study showed bands at 3332.16, 2114.20, 1635.70 and 637.27 cm^−1^. The broad band at 3332.16 cm^−1^ is attributed mainly to O—H stretching of hydroxyl-containing phytochemicals, with possible contribution from N—H groups, whereas the prominent band at 1635.70 cm^−1^ is associated with amide-I/carbonyl and/or C=C stretching vibrations. These functional groups are consistent with the presence of phenolic, flavonoid and protein-associated constituents of the plant extract and support their possible involvement in reduction and surface stabilization of the AgNPs. This result explains the ability of the biomolecules coating the nanoparticles, which prevents from agglomeration and stabilizes the nanoparticle. The FTIR spectroscopic analysis attests to the synergistic extract's capacity to carry out the dual tasks of stabilizing and reducing silver nanoparticles. Fig. 4 shows the spectrum of the silver nanoparticles synthesized by the synergistic herbal formulation of *Cassia fistula* and *Manilkara zapota.*^43^

**Fig. 4:**
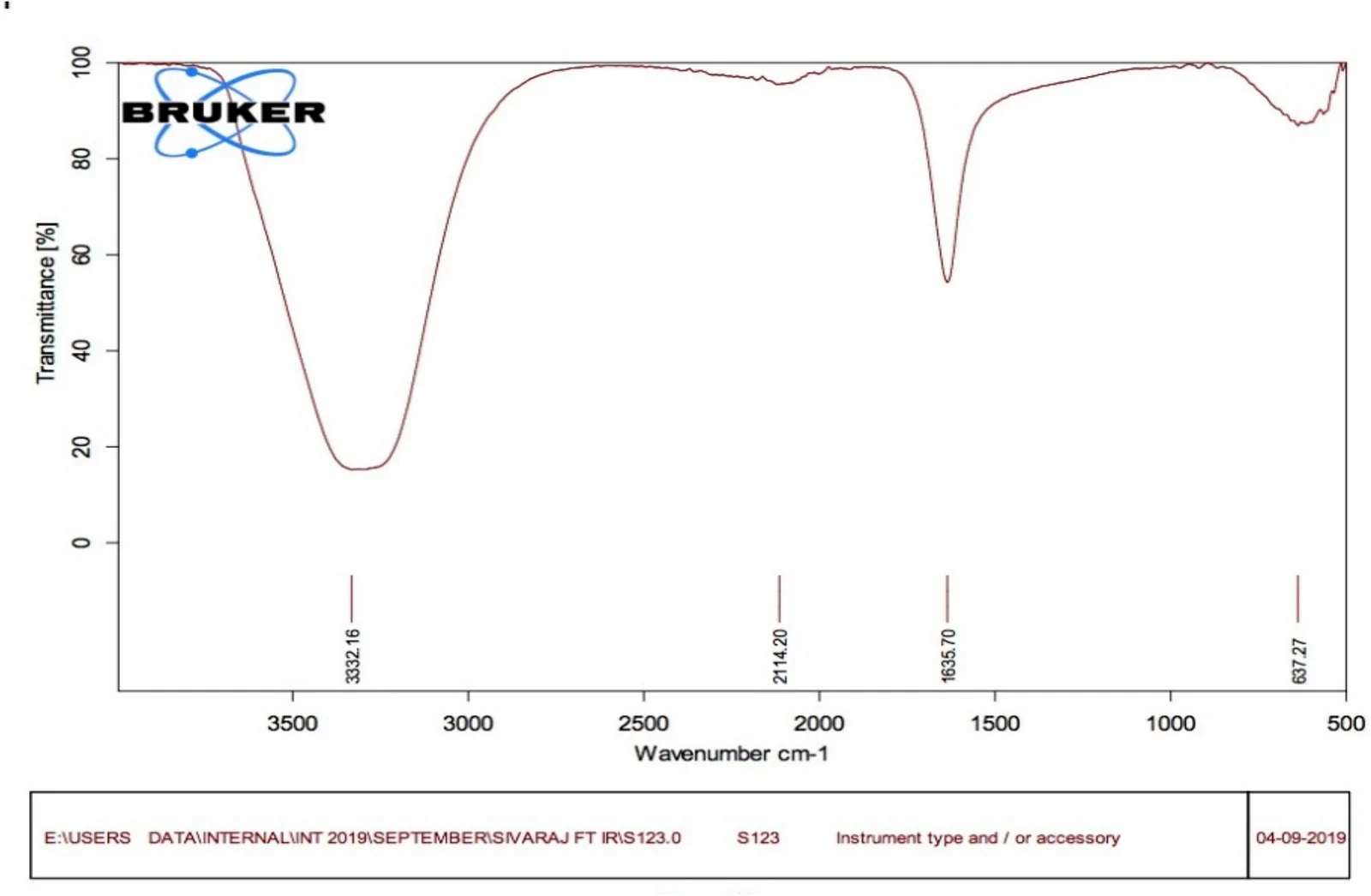
FTIR Spectrum of silver nanoparticles synthesized by methanol extract of *Cassia fistula* and *Manilkara zapota.*

The antibacterial screening of the silver nanoparticles in opposition to *Staphylococcus aureus* (*MTCC 96*) was performed by Disc diffusion method. Table 1, shows the maximum zone of retardation at 17 mm for *Staphylococcus aureus.* Fig. 5 shows the zone of inhibition of the extract. In comparison to the other microbes, the highest zone of retardation against *Staphylococcus aureus* was demonstrated by the manufacture of silver nanoparticles by *Cassia fistula.*^44^ Thus, the synergistic plant AgNPs provides the best bactericidal effect and provides less wound healing time than the other drugs. These studies were in accordance with the former studies that examined the similar concept.

**Table 1:** Antibacterial activity of poly herbal silver nanoparticles (1:1:1) against *Staphylococcus aureus.*

Pathogen	Concentration / Zone of inhibition (mm)				
	250 μg/mL	500 μg/mL	750 μg/mL	1000 μg/mL	Standard 25 μL (Tetracycline)
*Staphylococcus aureus*	13	15	16	17	14

**Fig. 5:**
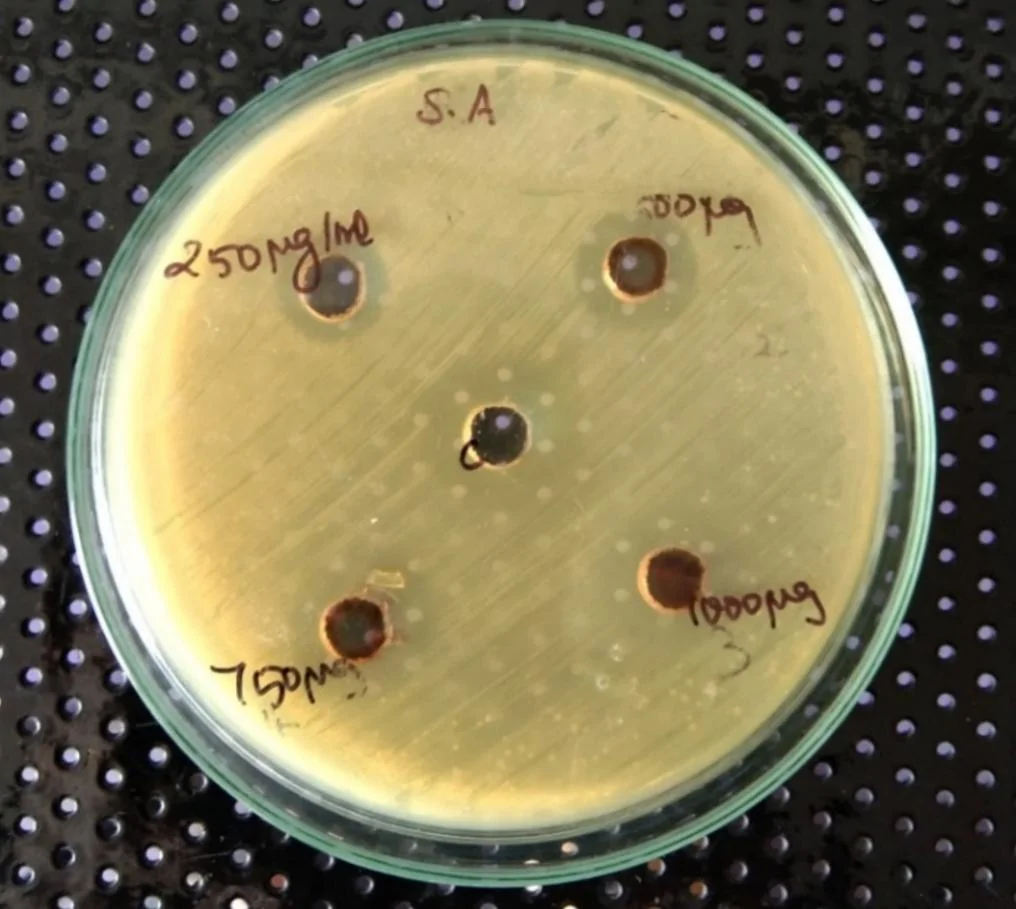
Zone of inhibition of plant silver nanoparticle against *Staphylococcus aureus.*

Previous studies of silver nanoparticles synthesized using *Cassia fistula* leaf extract have characterized the particles by UV–visible, FT-IR, SEM, EDX and AFM and reported antibacterial activity against several pathogenic microorganisms. Green-synthesized silver nanoparticles have also been investigated for antibacterial, antioxidant and anti-inflammatory applications.^45^ Silver nanoparticles synthesized from *Cassia fistula* bark extract have likewise been reported to possess notable antidiabetic activity in addition to their antimicrobial properties.^46^ Due to their vital functions, the silver nanoparticles synthesized by the synergistic plant extract were used as bactericidal, anti-cancerous and anti-inflammatory in various medical applications. This led to large-scale synthesis of nanomaterials used in wound healing and other biomedical applications. The silver nanoparticles along with the synergistic plant extract, would have worked vitally as an extra boost to our concept. With these results, further research will be carried out using these synergised plant-based silver nanoparticles in health care or translational medicines. *S. aureus* is a major pathogen associated with skin and soft-tissue infections and is frequently implicated in wound infections. It is also a normal colonizer of human skin and can become an opportunistic pathogen following disruption of the skin barrier. The PSN formulation produced a 17 mm inhibitory zone towards *Staphylococcus aureus* (MTCC 96) at the highest concentration tested. This is comparable to or exceeds several green-synthesis research studies; Jafari Sales & Shariat documented approximately 14 mm zones for eucalyptus-derived Ag NPs under similar disc diffusion conditions.^22^ The relatively larger inhibition zone found in our study can be attributed to (i) the enhanced availability of Ag^+^ ions due to the specific peak of polyphenols and proteins from the combined extracted substances, (ii) the prospect of synergistic antimicrobial effects associated with phytochemicals from plants (flavonoids, tannins) combined with the Ag NPs, and (iii) differences in the experimental conditions (NP concentration used on the disc, agar depth, bacterial culture, and incubation duration). Previous studies have demonstrated increased activity of Ag NPs sourced from extracts rich in phenolics, corroborating the hypothesis that extract composition affect's bactericidal ability.^21^, ^44^ Silver nanoparticles can exert antibacterial effects through interactions of released Ag^+^ ions and nanoparticle surfaces with bacterial cell membranes and cellular components, leading to membrane damage and disruption of cellular functions.^24^, ^45^ This antibacterial effect stops secondary infections from happening at the wound site, keeping the area clean and safe for healing.^44^

### 3.1 Antioxidant assay

Methanol extracts of *Cassia fistula* and *Manilkara zapota* contain abundant polyphenols (flavonoids and tannins) recognized for free-radical scavenging properties. These compounds counteract reactive oxygen species (ROS) generated during the inflammatory phase of wound healing, mitigating cellular oxidative stress. This protective mechanism aligns with the observed reduction in lipid peroxidation (LPO) and the concurrent elevation of superoxide dismutase (SOD) and catalase (CAT) activities in treated animals (Table 2).

**Table 2:** Comparison in level of LPO, SOD and CAT in skin tissues of SD rats when treated with standard ointment and PSN.

Sl.no	Parameter	Group I	Group II	Group III	Group IV
1	LPO (nmoles/min/mg protein)	3.12 **±** 0.16	1.97 **±** 0.15	1.32 **±** 0.12	1.26 **±** 0.25
2	SOD (nmoles/min/mg protein)	10.52 **±** 0.11	18.13 **±** 0.64	24.13 **±** 0.31	25.11 **±** 0.23*
3	CAT (nmol/mg protein)	18.32 **±** 0.51	21.28 **±** 0.70	27.22 **±** 0.20	29.10 **±** 0.21*

In Group I (untreated negative control) and Group II (povidone iodine), higher LPO levels (3.12 ± 0.16 and 1.97 ± 0.15 nnmoles/min/mg, respectively) indicated persistent oxidative stress. In contrast, Groups III (PCE) and IV (PSN) maintained normal, lower peroxidation levels (1.32 ± 0.12 and 1.26 ± 0.25 nnmoles/min/mg), preserving cell viability.

Antioxidants were substances that protected the cells from damage and other diseases. Antioxidants were mainly present in all plants and parts of the plants. It seems polyphenols were the most abundant antioxidants present in the plants. The plants which we used for study contains polyphenols like flavonoids and tannins which were widely investigated to prevent and treat from oxidative damage. The normal peroxidation level in the normal tissue is 1.75 nmoles per of melonialdehyde/g of tissue.^47^

Enzymatic defense markers showed statistically significant, dose-dependent increases on day 14 (*p* < 0.01):

• **SOD Activity:** Group IV (PSN) demonstrated the highest level (25.11 ± 0.23 nnmoles/min/mg), representing a 2.38-fold increase over Group I, a 2.29-fold increase over Group III, and a 1.72-fold increase over Group II.
• **CAT Activity:** Group IV led with 29.10 ± 0.21 nmol/mg, yielding a 1.58-fold increase over Group I, a 1.48-fold increase over Group III, and a 1.16-fold increase over Group II.

These findings demonstrate that the high phenolic content of the polyherbal nano-solution effectively accelerates wound healing by reducing systemic oxidative stress at the injury site.^4^, ^7^

Green-synthesized silver-based nanoparticles have also been reported to exhibit antioxidant activity in addition to their other biomedical effects.^15^, ^45^ However, the present findings are consistent with improved antioxidant status rather than directly antioxidant mechanism. These compounds counteract reactive oxygen species (ROS) generated during the inflammation stage of wound healing, consequently diminishing oxidative stress and reducing damage to cells.^28^ This mechanism aligns with the observed elevation in SOD and CAT enzyme activity and the reduction in the lipid peroxidation process in PSN-treated animals.

Group I: negative control, Group II: rats treated with povidone iodine, Group III: rats treated with PCE and Group IV: rats treated with PSN.

In the Table 2, the degradation of lipid level in terms of LPO was seem to be normal in group III and IV compared to the group I and II skin tissue of rats. The peroxidation level is higher in group I and group II which leads to oxidative stress. The medical formulation which inhibits the lipid peroxidation were trusted to increase the viability of the cells which promotes and prevents the cellular damage. One of the antioxidant parameters which was measured was the superoxide dismutase. This assay showed us the significant increase with respect of the treatment groups. In Table 2, the SOD level in was higher in group IV than the group II and group I animals. Reported that the SOD was much needed for accelerating the wound healing, there was 2.38-fold increase between group I and group IV,2.29-fold increase between group I and group III and 1.72-fold increase between group I and group II with *p* < 0.01 level of significance. One antioxidant enzyme called SOD sought out the superoxide anion by transforming free radicals into oxygen and hydrogen peroxide, thereby averting damages brought on by free radicals. Because of its scavenging ability, SOD was the most significant enzyme in therapeutic purposes. Catalase was a common enzyme found on all living organisms which was exposed to oxygen and catalysed the decomposition of hydrogen peroxide to oxygen and water. It was a very important enzyme which was used to protect the damage by ROS. This enzyme was mainly present in all the fruits and vegetables. Our plant sources which were high in phenolic content reported the treatment against oxidative damage after 14 days of wound healing treatment in different group of rats. The catalase showed a significant increase in Table 2 compared to the control and the standard group of rats. There was 1.58-fold increase between group I and group IV, 1.48-fold increase between group I and group III and 1.16-fold increase between group I and group II with * *P* < 0.01 level of significance. Thus, the high phenolic content in the plants which was chosen, leaded to high flavonoid and tannin content that helped in accelerating the wound healing by reducing the stress. Significant increase in the catalase enzyme level of plant extract of *Cassia fistula* treated rats, reported that, it gives high flavonoid and phenolic content that can control the stress level of the individuals. Therefore, the antioxidant enzymes from the assay findings depicted the potentiality of the antioxidants in the polyherbal silver nano solution and its attributes towards the wound treatment. With the reference works on these studies, we also conclude that the early secretion of these enzymes and its elevation level do help the stages of wound healing.

### 3.2 In vitro scratch assay

The scratch assay was utilized to qualitatively evaluate fibroblast migration and proliferation across a fixed observation window (0 to 72 h) using 200 \(\mu \)g/mL and 300 \(\mu \)g/mL PSN concentrations (Fig. 6).

**Fig. 6:**
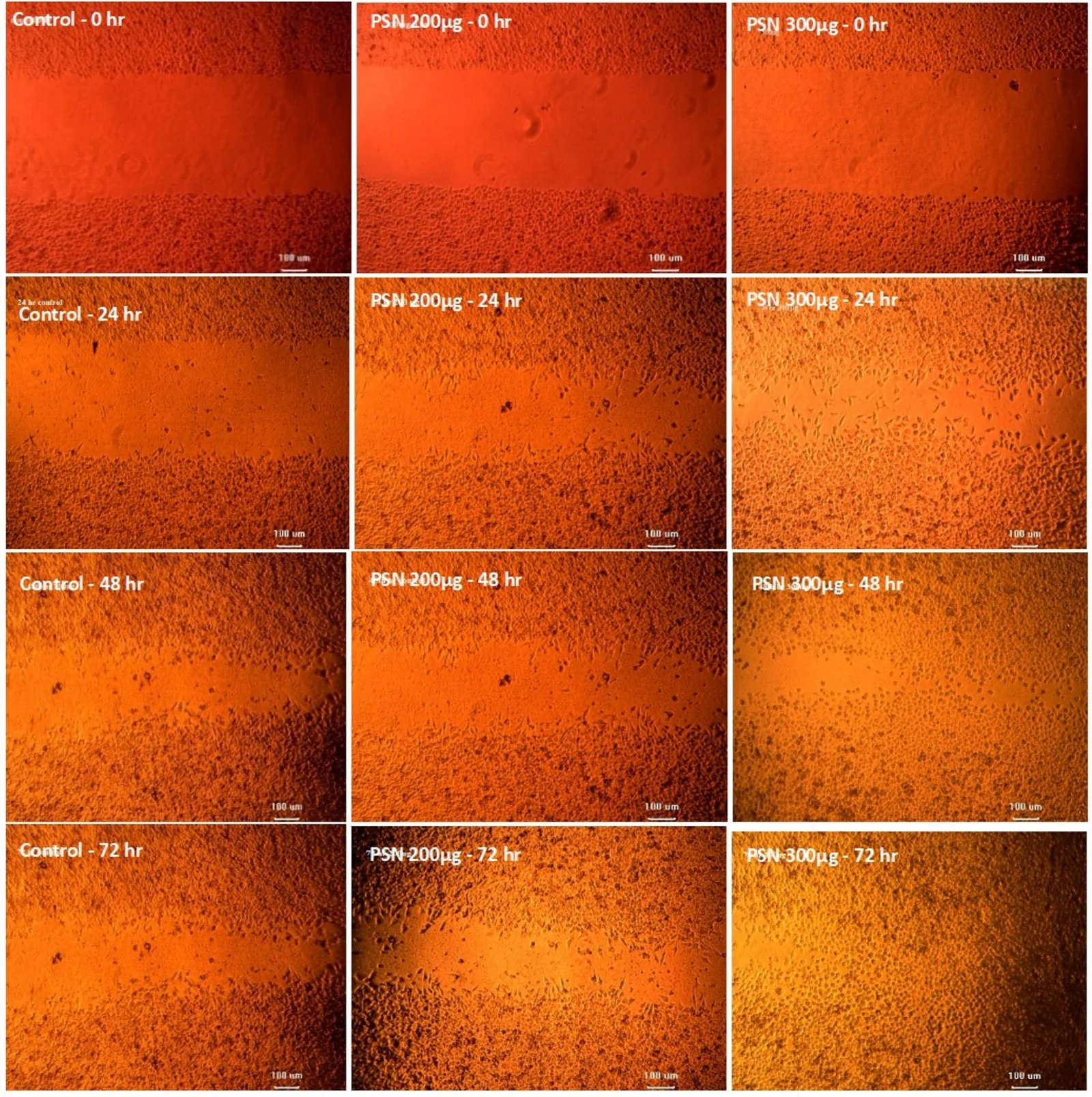
Migration of fibroblasts cell line treated with povidone iodine ointment and PSN at 200 μg/ml and 300 μg/ml with its graphical representation.

Both PSN groups accelerated scratch closure compared to the control group by inducing progressive cell monolayer movement into the boundary gaps. By hour 72, full re-epithelialization was achieved in the PSN-treated layers. This enhanced cellular migration is consistent with literature indicating that flavonoid-capped silver nanoparticles upregulate localized growth factors (such as VEGF and TGF-\(\beta \)), which actively stimulate keratinocyte and fibroblast proliferation.

Cell migration and proliferation played the major role in wound healing. The scratch assay had been used to study the migration attributes of the fibroblasts cell lines treated with PSN. The wound healing concentration was fixed to 200 μg/ml and 300 μg/ml of PSN formulation. Incubation period was fixed from the 0th hr to 72 h. Fig. 6 showed the scratch of the cell line at the 0th hr,24th hr,48th hr and 72nd hr respectively. Each picture had an increase in the proliferation of the cell line compared to the control by closing the gaps of the scratch. At the 72nd hr, there was full epithelization of the layer. This reported us that the PSN formulation accelerated the wound contraction where the cell mono layer moved towards the scratch and fastens the wound contractions (Fig. 7). Control rats showed the delayed wound contraction compared to the polyherbal silver nanoparticle formulation. The PSN formulation improved the movement of fibroblasts, as shown by the in vitro scratch test, which suggests that it encourages cell growth, these findings are interpreted as qualitative evidence of enhanced cellular migration. Silver nanoparticles have been documented to enhance the expression of growth factors, including VEGF and TGF-β, which activate fibroblast and keratinocyte functions, resulting in expedited re-epithelialization and collagen production.^48^, ^49^ Flavonoid-capped silver nanoparticles have similarly been shown to enhance fibroblast migration in scratch wound assays, supporting their role as promising wound-healing agents.^50^

**Fig. 7:**
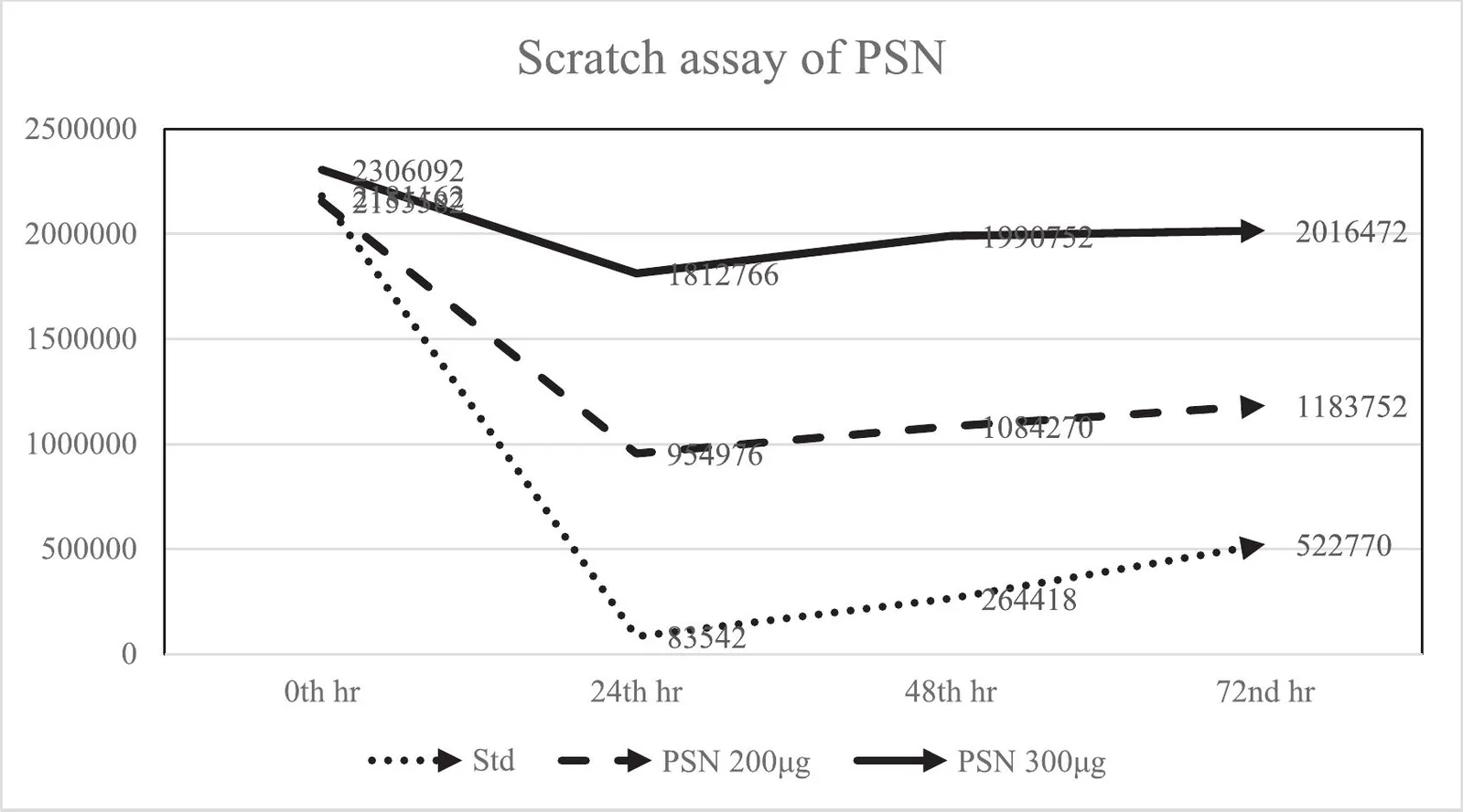
Graphical representation of the scratch assay.

### 3.3 Evaluation of excision wound model

Topical applications were evaluated using a surgical excision model over 14 days (Fig. 8, Table 3). Group IV (5% *w*/w PSN ointment) demonstrated optimal healing kinetics, showing a statistically significant expansion in wound contraction rates compared to all other cohorts (*p* < 0.001):

• Day 2 Initial Response: Group IV achieved an immediate 38.57 ± 0.43% contraction, while Group III (PCE) reached 36.20 ± 0.54%, vastly outperforming Group II (3.29 ± 1.20%) and Group I (0.32 ± 1.52%).
• Day 14 Final Closure: Group IV reached 75.58 ± 0.12% closure. This represents a 1.30-fold increase over Group I (57.88 ± 0.37%), a 1.21-fold increase over Group III (70.39 ± 0.14%), and a 1.17-fold increase over standard Group II povidone‑iodine (64.11 ± 0.17%).

**Fig. 8:**
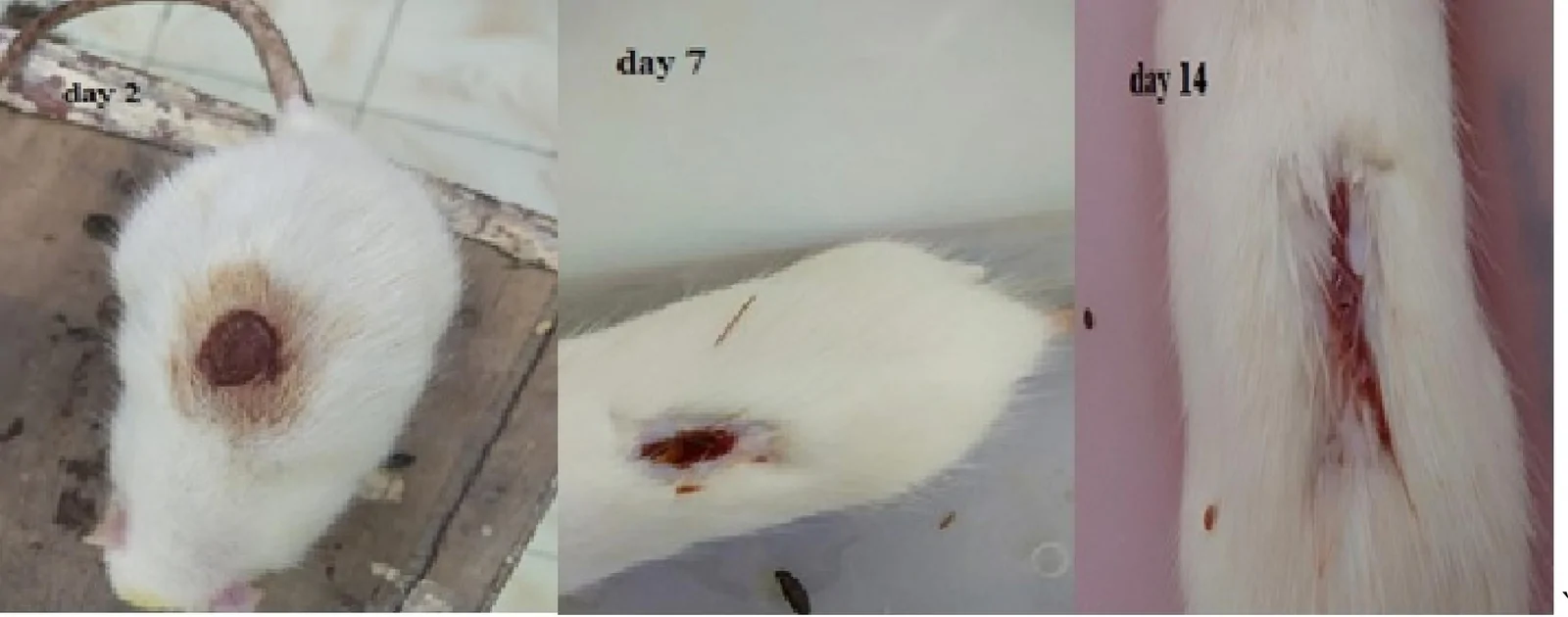
Pictures of group IV rats, epithelization, and wound contraction representation on day 2,7 and 14.

**Table 3:** Effect of wound healing of rats treated with PSN and PCE in comparison with the povidone iodine treated rats.

Day	Negative control Group I %	Standard ointment Group II %	PCE Group III %	PSN Group IV %
2	0.32 ± 1.52	3.29± 1.20	36.20 ± 0.54	38.57 ± 0.43*
4	10.04± 1.20	27.98± 0.55	43.93± 0.36	44.67 ± 0.23*
6	19.26 ± 1.43	34.60± 0.73	44.67± 0.28	46.15± 0.14*
8	27.98 ± 0.78	51.16± 0.64	59.81± 0.22	66.47 ± 0.13*
10	43.93 ± 0.45	52.54± 0.55	69.85± 0.24	70.39 ± 0.13*
12	51.15 ± 0.46	57.88± 0.37	70.93± 0.24	73.05 ± 0.14*
14	57.88 ± 0.37	64.11 ± 0.17	70.39 ± 0.14	75.58 ± 0.12*

Wounds were observed on days 2,4,6,8,10,12 and 14 after wound creation. The percentage of original wound area were shown for the above days. Each column represented the mean ± SD. The significance values were * *p* < 0.001 representing the value comparison between group I, group II, group III, and group IV.

The wound contraction of group II was 1.10-fold increased than group I, whereas the contraction rate of group III was 1.21-fold increase than the group I. Group IV showed a contraction rate of 1.3-fold increase. Group IV showed 1.17-fold increase than the group II rats, thus proving PSN to be the efficient natural medicine for the wound healing.

The high efficiency of the PSN formulation is attributed to dual-action synergy: biogenic silver ions offer robust antimicrobial protection to prevent secondary infections, while plant-derived bio-reductants lower local oxidative profiles to facilitate rapid tissue reconstruction.

Medicine of 50 μM VCN-2 film gave the highest wound contraction percentage of 39.91 ± 3.17% on day 7 and 63.06 ± 1.94% on day 14. The allantoin film-treated positive control rats showed encouraging recovery outcomes, with a wound contraction percentage of 59.35 ± 2.03% throughout 14 days of treatment. Polyherbal silver nanoparticle showed a good wound healing effect in normal rats compared to the diabetic rats which was treated with the Vicenin −2 sodium alginate, which was a synthetic formulation for the diabetic rats. Thus, the results confirmed and suggests that the herbal topical medication with the nano technique possess decent healing potential than the other topical applications.^51^

A wound healing model using mice. SD rats had wounds made in their backs. Wounds were observed on days 2, 7 and 14 after wound creation.

### 3.4 Histological evaluation of wound tissue

Microscopic examination of excised skin tissue on day 14 provided qualitative histopathological evidence of structural repair (Fig. 9).

**Fig. 9:**
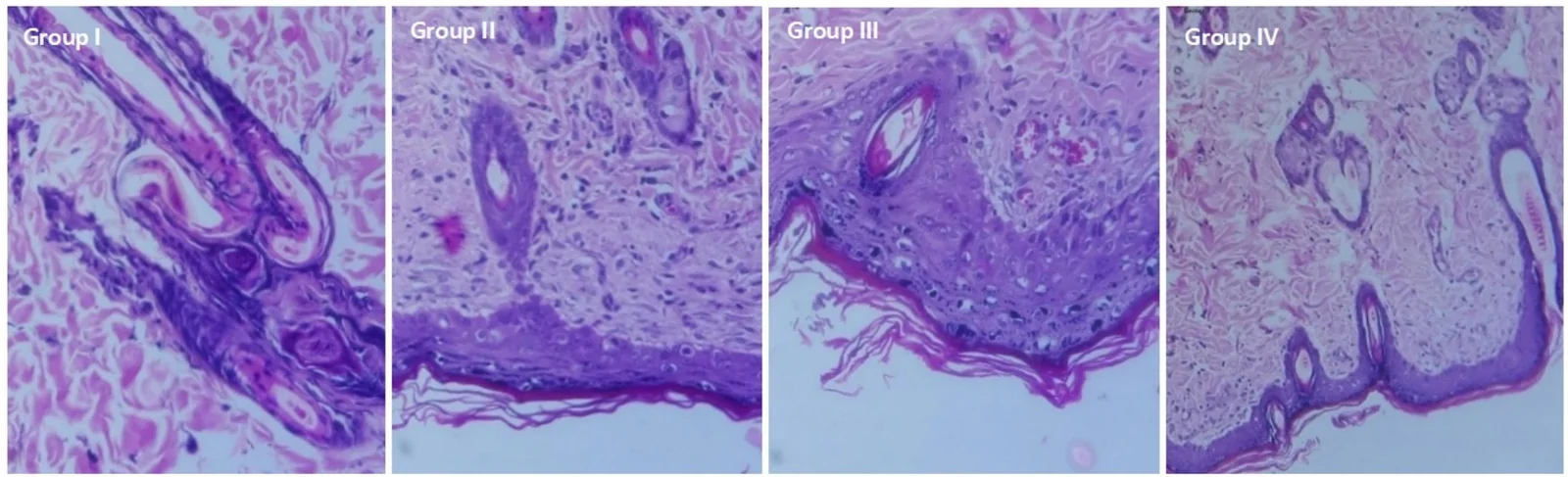
Histological examination of group I, II, III and IV rats on *Re*-epithelization and tissue repair of the skin.

Untreated and standard-treated groups showed incomplete epidermal reconstruction. In contrast, Group IV (PSN-treated) tissue demonstrated accelerated matrix remodeling, characterized by extensive fibroplasia, robust angiogenesis, dense collagen deposition, and advanced, uniform re-epithelialization across the wound bed.

Histological evaluation should contain the components like angiogenesis, inflammation, fibroplasia and restoration of the connective tissue matrix, wound contraction and remodeling, epithelialization, and differentiation. In the present study, histopathological evaluation was performed as a qualitative assessment of the wound-healing features observed microscopically. On the 14th day of the wound treatment, rat tissues were examined for the evaluation of the healing process. On the 14th day, the whole bed was filled with collagen and inflammatory cells and improved re-epithelization took place. In Fig. 9, PSN treated group IV rats comparatively had good re-epithelization forming a bed like structure than the other groups of treated rats. This reported that the re-epithelization had started early compared to others. As this evaluation was qualitative and no formal semi-quantitative histopathological scoring system was applied, these findings are interpreted as qualitative histopathological observations.This study concluded that the PSN had high wound healing property than the control and the standard group of rats.^52^

Histopathology of the rat tissue explaining the re-epithelization of the skin at 540 μm. a – group I, b – group II, c – group III and d – group IV. Group IV PSN treated showed the acceleration of the wound healing by proper formation of the cell bed and re-epithelization.

### 3.5 Effect of TNF-α administration on wound healing

Serum TNF-\(\alpha \) concentrations were measured via ELISA on day 14 to assess late-stage inflammatory status (Table 4).Because TNF-\(\alpha \) is a pro-inflammatory cytokine typically released early by neutrophils and macrophages during the acute phase of wounding, its concentration progressively drops as successful wound contraction advances. On day 14, Group IV (PSN) displayed the lowest residual concentration at 6.49 ± 0.01 pg/mg of protein, followed by Group III at 8.76 ± 0.01 pg/mg. Both plant-derived formulations exhibited lower systemic inflammation than standard Group II (12.69 ± 0.02 pg/mg) (*p* < 0.01).This single late-stage measurement confirms that the dual-action polyherbal silver nanoparticles efficiently shorten the inflammatory phase, driving the wound cascade rapidly forward into the proliferative and regenerative stages.

**Table 4:** Effect of TNF – α administration in blood serum on wound healing.

Samples	Concentration of TNF- α from the std graph pg/mg of protein
Group I	5.78 ± 0.02
Group II	12.69 ± 0.02
Group III	8.76 ± 0.01
Group IV	6.49 ± 0.01

The interpretation was therefore moderated, and it was clarified that the present findings represent a single late-stage measurement of TNF-α rather than a complete time-course assessment of TNF-α during the wound-healing process. From these results and the wound contraction rate, we reported that the TNF- α cytokine was produced in higher concentration and in higher rates in the earlier wound healing stage, that was on the third or the fourth day of the treatment. It gradually decreased as there was an increase in wound contraction rate.^48^

The antioxidant nature of the plants on the wound were totally dependent on the phenolic compounds that were the phytochemicals which were present in the plants. Tannins, flavonoids and the saponins were the compounds present in our plants which improved the antioxidant potential of the formulation thus reducing the stress factor during the wound and wound healing stages. Biochemical parameters were one of the significant parameters to be checked for the medication on the wounds because any change in the levels of the protein in the body due external application of the medicine would cause severe damage to the tissues as well as the organs. The in vivo and the in vitro assays promised that the wound healing with the PSN were effective comparative to the chemical ointments. Thus, the crude polyherbal extract and the polyherbal silver nanoparticles exhibited the wound healing activity in rats.^49^

The statistical significance considered for the TNF – α secretion in blood serum by one way ANOVA calculation, *n* = 6 and mean ± SEM. The significance values were * *p* < 0.01 representing the value comparison between group I, II, III and IV. This showed that the cytokine tumour necrosis factor was an inflammatory protein and secretes higher during the initial period of the wound healing and decreases gradually (Mi et al., 2022). Thereby day 14 (Group IV), our applied PSN ointment had caused about 75.6% of the wound to close, which is better than what has been reported for other formulations. For instance, various topical nano formulations and herbal ointments exhibit wound contraction rates ranging from 60% to 65% at similar time points (e.g., allantoin film or specific nano-based positive controls.^21^, ^51^, ^53^ The significant contraction observed in our PSN group is likely due to the formulation's synergistic antimicrobial, antioxidant, and cell-proliferative properties. Ag NPs reduce the microbial load at the wound site, while plant phytochemicals decrease reactive oxygen species and enhance fibroblast migration and collagen synthesis, thereby accelerating re-epithelialization.^33^, ^49^ Differences in methodology (animal strain, wound size, ointment composition, nanoparticle loading, and determining techniques) could explain the differences in % contraction between studies. Hydroxyproline, collagen deposition, VEGF, TGF-β, and angiogenesis markers could provide additional mechanistic evidence; however, these markers were not measured in the present study and will be evaluated in future studies. The combination of metal-based AgNPs with phytochemicals improves the healing of wounds by working in two ways: Ag^+^ ions protect against bacteria while plant metabolites provide antioxidant and regenerative support. This combination leads to faster contraction, better formation of the epithelial layer, and lower levels of TNF-α, which shows that the inflammatory phase is shorter. The current study aimed to investigate the synergistic potential of combining extracts from *Cassia fistula* and *Manilkara zapota* in a green production of silver nanoparticles (PSN formulation). *Cassia fistula* includes flavonoids, tannins, and anthraquinones that are known for helping tissues heal and reducing inflammation. *Manilkara zapota*, on the other hand, is high in polyphenols and triterpenoids that are good for your health and kill bacteria.^1^, ^4^ When incorporated, the phytochemicals from the two sources serve as bio reductants and stabilizing agents in nanoparticle fabrication, resulting to the development of stable, well-capped AgNPs with improved surface reactivity. The mixed extract probably has a wider range of reducing and capping agents, like phenolic hydroxyls, carboxyls, and amine groups, that keep nanoparticles from getting too big and clumping together. This biochemical diversity also encompasses biological activity, wherein various classes of substances (flavonoids from *Cassia fistula* and saponins from *Manilkara zapota*) may synergistically facilitate accelerated epithelial regeneration, diminish oxidative stress, and strengthen antimicrobial defense. Previous reports have documented phytochemical synergy in other polyherbal nanoparticle formulations that surpass single-plant counterparts by modulating multiple pathways of healing cascades.^2^, ^20^ In our instance, the enhanced wound contraction (75.58% on day 14) and reduced TNF-α secretion signify this synergistic biological efficacy.

## 4 Conclusion

The current work explained about the preparation of the silver nanoparticle with the help of the polyherbal plant extract of *Cassia fistula* leaves and flowers and the *Manilkara zapota* leaves. This synthesis of silver nanoparticles with the synergistic plants created an effective SNP for our future wound studies. The objective was to assess the overall wound-healing potential of the plant-mediated AgNP formulation rather than to compare different synthesis routes. Wound healing studies exhibited at a concentration of 5%*w*/w of the polyherbal nanoparticle solution. The characterisation methods of nanoparticles like UV - Visible spectroscopy, SEM analysis and FT-IR confirmed that our synergistic silver nanoparticle was a stabilised nanoparticle which can be further used for various applications. The antibacterial activity of the synergistic silver nanoparticle by disc diffusion method justifies our work in resisting and killing the microbe which is responsible for wound and other related studies. Silver nanoparticle supported the plant extracts and decreased the excision wound within 8 to 9 days. The observations from the invitro assay, histopathological studies enhanced the hope of using the polyherbal silver nano solution as the future wound healing dressing to treat the wounds. The antioxidant assay also revealed that the stress level which is caused due to the wounds are reduced by the anti-oxidants present in the plant extracts which suppresses the oxidation process. Thus, this polyherbal silver nanoparticle could have the future applications in the development of the wound dressing as a promising biomaterial in patients for wound healing.

The study showed that the polyherbal silver nanoparticle formulation had a lot of promise for healing wounds, but with this work is that the botanical extracts of *Cassia fistula* and *Manilkara zapota* were not investigated on their own. Thus, while the increased efficacy indicates synergism, the precise quantitative impact of each extract remains indeterminate. Subsequent research should entail a comparative assessment of individual and mixed formulations to validate and quantify the extent of synergy. Systemic haematological, hepatic and renal safety profiling of the synergistic AgNP formulation, as has been performed for other biosynthesized silver nanoparticles, would also be a valuable direction for future work.^54^

## Consent for publication of individual participant data

Not applicable. No individual participant data were collected or reported.

## Human ethics statement

Not applicable. No human participants, human tissues, or human-derived biological samples were involved in this study.

## Generative AI and AI-assisted technologies

The authors declare that no generative artificial intelligence or AI-assisted technologies were used in the generation of scientific data, analysis, interpretation, or conclusions presented in this manuscript. If applicable, AI-assisted tools were used only for language editing and improvement of English readability, with the authors retaining full responsibility for the content.

## Declaration of originality

The authors declare that this manuscript is an original work, has not been published previously, and is not currently under consideration for publication elsewhere. All authors have approved the manuscript and its submission to the journal.

## CRediT authorship contribution statement

**Archana Hari:** Writing – review & editing, Writing – original draft, Software, Methodology, Conceptualization. **Shobana Sampath:** Writing – review & editing, Writing – original draft, Formal analysis, Conceptualization. **B. Vijayageetha:** Writing – review & editing, Writing – original draft, Software, Resources, Methodology, Conceptualization. **Douglas Law:** Writing – original draft, Software, Resources, Methodology, Investigation, Conceptualization. **Gholamreza Abdi:** Writing – review & editing, Writing – original draft, Supervision, Project administration, Conceptualization.

## Consent to participate

Not applicable. No human participants were involved in this study.

## Animal ethics statement

Not applicable. No experimental animals were used in this study.

## Clinical trial registration

Not applicable. This study did not involve a clinical trial.

## Ethical approval

This study was carried out in accordance with the Institutional Animal Ethics Committee, (IAEC) of C.L. Baid Metha College of Pharmacy, Chennai, Tamil Nadu, with approval No: 06/321/PO/Re/S/01/CPCSEA/dated 31/03/19.

## Author agreement

All authors have read and approved the final manuscript and agree with its submission for publication. The authors confirm that they have contributed substantially to the work and accept responsibility for the integrity and accuracy of the research.

## Funding

The authors received no specific financial support or funding from any public, commercial, or not-for-profit funding agency for this research.

## Declaration of competing interest

The authors declare that they have no known competing financial interests or personal relationships that could have appeared to influence the work reported in this paper.

## Data Availability

All data generated or analysed during this study are included in this published article. The raw datasets supporting the findings of this study are available from the corresponding author on reasonable request.
